# Optimization of Carbon Ion Treatment Plans by Integrating Tissue Specific α/β-Values for Patients with Non-Resectable Pancreatic Cancer

**DOI:** 10.1371/journal.pone.0164473

**Published:** 2016-10-13

**Authors:** Constantin Dreher, Christian Scholz, Mira Pommer, Stephan Brons, Hannah Prokesch, Swantje Ecker, Jürgen Debus, Oliver Jäkel, Stephanie E. Combs, Daniel Habermehl

**Affiliations:** 1 Department of Radiation Oncology, University Hospital Heidelberg, Heidelberg, Germany; 2 Department of Radiooncology, Klinikum rechts der Isar, Munich, Germany; 3 Imaging & Therapy Division, Healthcare Sector, Siemens AG, Mannheim, Germany; 4 Hottinger Baldwin Messtechnik GmbH, Darmstadt, Germany; 5 Heidelberg Ion Beam Therapy Center (HIT), University Hospital Heidelberg, Heidelberg, Germany; 6 Institute of Innovative Radiotherapy (iRT), Helmholtz Zentrum München, München, Germany; North Shore Long Island Jewish Health System, UNITED STATES

## Abstract

**Background:**

The aim of the thesis is to improve treatment plans of carbon ion irradiation by integrating the tissues’ specific αβ-values for patients with locally advanced pancreatic cancer (LAPC).

**Material and Methods:**

Five patients with LAPC were included in this study. By the use of the treatment planning system *Syngo RT Planning* (Siemens, Erlangen, Germany) treatment plans with carbon ion beams have been created. Dose calculation was based on αβ-values for both organs at risk (OAR) and the tumor. Twenty-five treatment plans and thirty-five forward calculations were created. With reference to the anatomy five field configurations were included. Single Beam Optimization (SBO) and Intensity Modulated Particle Therapy (IMPT) were used for optimization. The plans were analyzed with respect to both dose distributions and individual anatomy. The plans were evaluated using a customized index.

**Results:**

With regard to the target, a field setup with one single posterior field achieves the highest score in our index. Field setups made up of three fields achieve good results in OAR sparing. Nevertheless, the field setup with one field is superior in complex topographic conditions. But, allocating an αβ-value of 2 Gy to the spinal cord leads to critical high maximum doses in the spinal cord. The evaluation of dose profiles showed significant dose peaks at borders of the αβ-gradient, especially in case of a single posterior field.

**Conclusion:**

Optimization with specific αβ-values allows a more accurate view on dose distribution than previously. A field setup with one single posterior field achieves good results in case of difficult topographic conditions, but leads to high maximum doses to the spinal cord. So, field setups with multiple fields seem to be more adequate in case of LAPC, being surrounded by highly radiosensitive normal tissues.

## Introduction

About 277,000 people worldwide die each year due to pancreatic cancer—these patients are still having a dismal prognosis [1]. Currently, resection is the only curative treatment. In case of non-resectable disease neoadjuvant treatment approaches including combined chemoradiation with gemcitabine have proven efficacy towards tumor downsizing and lead to a secondary resectability in approximately 30% [2]. Dose exposure to organs at risk (OAR) can be reduced significantly by the use of modern radiotherapy techniques such as IMRT (Intensity-Modulated Radiotherapy) and IGRT (Image-Guided Radiotherapy) [3].

Due to distinct physical and biological characteristics, particle therapy is able to offer an even more conformal dose delivery—a high dose deposition in the target volume and an increased sparing of the OARs. Proton and carbon ion radiotherapy are characterized by an inverted depth-dose-curve, which leads to a low dose deposition within the entry channel and a well-defined high local dose deposition in the Bragg Peak region [4]. Moreover, with an enhanced and prolonged DNA (deoxyribonucleic acid) damaging, carbon ions offer higher RBE (Relative Biological Effectiveness) values [5].

There are also encouraging clinical results from Japanese particle therapy facilities, that have conducted smaller clinical trials with pancreatic cancer patients and gained experience with carbon ion treatment using different treatment protocols over the last few years [6, 7]. Particle therapy of abdominal organs is a complex treatment because inter- and intra-individual changes in organ motion and bowel gas movement may have a serious impact on ion beam dosimetry [8, 9].

In a previous study our group focused on a comparison of different field setups–both for protons and carbon ions in patients with non-resectable pancreatic cancer (same dataset as in this manuscript) [10]. So far, research in treatment planning of ion beam therapy is generally based on a fixed RBE or αβ-value. The purpose of this study is to create carbon ion treatment plans for pancreatic cancer with tissue-assigned specific αβ-values. This study is a preparation for the clinical practice in the PHOENIX trial using active raster scanning technology [11, 12]. Optimization strategies based on αβ-values are developed and different carbon ion field configurations will be analyzed with regard to dose coverage of the target volume and dose sparing of the OARs using a recently introduced customized rating scheme.

## Material and Methods

### Patients’ and anatomical characteristics

The medical ethics commission of the medical faculty of Heidelberg has approved the presented in-silico study (S-483/2011). Five LAPC patients, being treated with photon radiotherapy at our institution, were included in this study. Treatment planning CT (computed tomography) scans were performed under free breathing, and both with and without contrast agent. Patients were immobilized in supine position.

In order to determine the impact of the cases’ topography, the minimal distance between two structures was measured in each horizontal slice over the total target volume extension, and afterwards the mean value has been calculated.

1. Mean Xmin kidney ri-le = mean minimum distance between both kidneys
2. Mean Xmin target-kidney left = mean minimum distance between target and left kidney
3. Mean Xmin target-kidney right = mean minimum distance between target and right kidney
4. OAR intersection = intersection between target and OARs

The anatomical and patients’ characteristics are summarized in Table 1.

**Table 1:** Patients’ and anatomical Characteristics.

Patients’ characteristics:	Case 1	Case 2	Case 3	Case 4	Case 5
**Gender**	Male	Male	Female	Male	Male
**Age at CT scan [years]**	71	77	64	67	67
**Cancer location**	Caput	Caput	Caput	Caput/Cor-pus	Caput/Cor-pus
**Target volume [cm**^**3**^**]**	333	92	166	224	150
**Anatomical characteristics:**					
**Mean Xmin kidney right-left [cm]**	10.1	9.6	7.5	10.6	7.5
**Mean Xmin target-kidney left [cm]**	5.4	6.0	3.1	5.3	4.1
**Mean Xmin target-kidney right [cm]**	3.2	3.2	3.6	2.5	2.5
**OAR Intersection**	• Large intestine	• Large intestine	• Large intestine • Stomach/ duodenum • Liver	• Large intestine	• Large intestine • Stomach/ duodenum • Liver

CT = computed tomography, Xmin = minimal distance, OAR = Organ at risk

### Target volume definition

The original treatment plans had a target volume made up of a PTV (planning target volume) including elective nodes and a boost volume including the GTV (gross tumor volume) and a margin of 2–4 mm at the discretion of the responsible specialist. These boost volumes are the target volumes in this study.

### Treatment Planning System

At HIT (Heidelberg Ion-beam Therapy Center) the inverse treatment planning system *Syngo RT Planning* (Siemens, Erlangen, Germany) is established. *Syngo RT Planning* is based on the effective dose calculation model LEM I (Local Effect Model I) as described by Krämer & Scholz [13].

### Treatment Planning System Optimization

Treatment planning is either possible by *single field uniform dose optimization* (SBO, Single Beam Optimization) or by *multiple field optimization* (IMPT, Intensity Modulated Particle Therapy). Both tools use intensity modulation. IMPT integrates all beams and optimizes simultaneously. SBO allows relative weighting factors to each beam. These beams are optimized independently and add up to 100% of the prescribed dose.

### Field Setup (FS)

**Fig 1:**
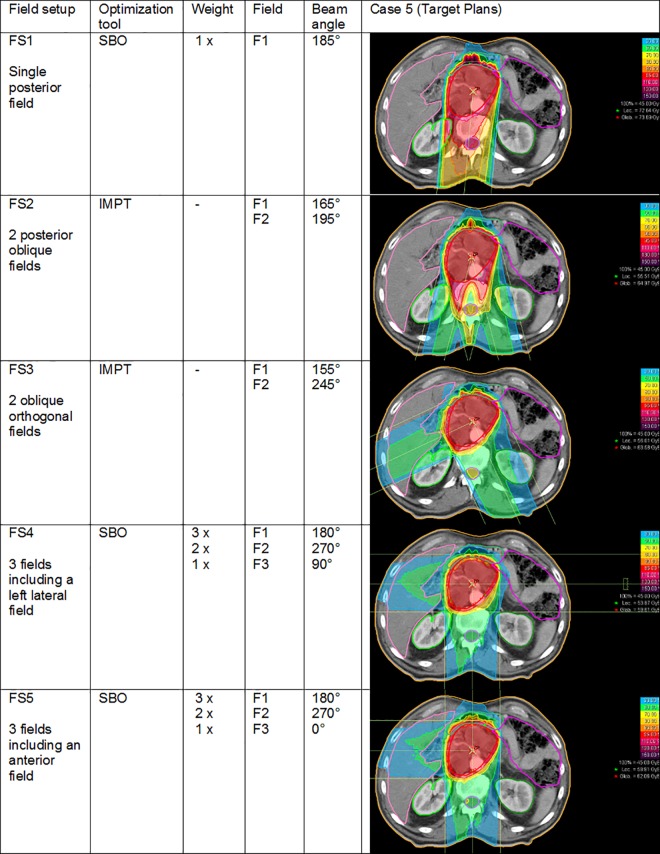
Field Setups: Characteristics. Description of 5 field setups with their dose distribution in case 5.

According to our former study, five different field setups (FS) at the gantry were considered to be relevant and used for all cases (Fig 1)—although slightly adapted to different topography [10]. Gantry beam angles are described according to the International Electrotechnical Commission (IEC).

The number of fields is restricted to 3 fields, which is due to two reasons: on the one hand the topographical conditions of pancreatic cancer, which is situated centrally in the abdomen. By increasing the number of fields the interfractional robustness of the field setup may be decreased because of the higher number of beams directed through inconsistent filling volumes of the bowel and the bowel’s movement, thus leading to an important impairment of treatment planning. On the other hand carbon ion radiotherapy is a very time consuming technique, which is why the number of fields is to be limited to a realistic level.

The beam angle of FS1 is mainly due to the short field length, reducing the dose exposure of normal tissues and the avoidness of varying penetration range due to motion or changing air cavities with anterior fields. FS2&3 try to keep hold of the good advantages of FS1 and to reduce the dose exposure of the spinal cord. For the FS made up of 3 fields the decision is mainly based on investigations of Kumagai et al, implementing these field configurations with little dose exposure to organs at risk but showing a decrease of robustness with anterior-posterior and lateral fields [8]. With SBO being advantageous in case of active restriction of a beam, this kind of optimization is used with field configurations including fields that can possibly result in less robustness (FS4 and FS5). Therefore, the greatest weight was given to the posterior field. By the use of SBO with FS4 and FS5 we can increase robustness. IMPT is possible with all fields located in regions of little anatomical variability.

### Dose prescription

At HIT a slightly hypofractioned dose regime with a single dose of 3 Gy(RBE) has been established for carbon ion irradiation. We chose a fraction number of 15 representing the second escalation dose in our forthcoming clinical PHOENIX trial on dose escalated carbon ion therapy for patients with pancreatic cancer [11]. The specific αβ-value of 4.5 Gy for pancreatic cancer has been identified in our own department as a preparation for the PHOENIX trial [11, 14]. For this study, which has to be regarded as a worst case scenario, the αβ-value was consciously rounded up to 5 Gy. The calculated total dose adjusted to the fractionation effect according to the linear-quadratic model and an αβ-ratio of 5 Gy results in approximately 51.4 Gy (BED 2Gy), which is effective for both neoadjuvant and definitive treatment protocols in non-resectable pancreatic cancer patients. The plans are scaled to the median dose of 45 Gy(RBE).

### α/β-values

αβ-values of the spinal cord, the kidneys, the liver, and the stomach are generally fixed to an αβ-value of 2 Gy, thus orientated to late tissue effects [15]. The skin’s αβ-values for late effects is set to 3 Gy [16, 17]. The αβ-values are summarized in Table 2. Biological dose calculation with these αβ -values has been verified by the TPS TrIP (Treatment Planning for Particles) [18].

**Table 2:** αβ ratios, Target, OAR and Cumulative criteria.

Structure	αβ-ratio	Constraints			Max. points	Sum of max. points	Score	
**Target:**	5.0 Gy	V44	≥	90.0%	10	40	Target Criteria	Cumulative Criteria
		1-V42.75	<	5.0%	10			
		V50	<	1.0%	10			
		Min	>	40.0 Gy(RBE)	10			
**Spinal cord:**	2.0 Gy	Max	<	24.0 Gy(RBE)	15	15	OAR Criteria	
**Each Kidney:**	2.0 Gy	V15	<	15.0%	5	15		
		D25	<	10.0 Gy(RBE)	5			
		Mean	<	12.0 Gy(RBE)	5			
**Liver:**	2.0 Gy	V20	<	12.5%	5	15		
		V10	<	20.0%	5			
		Mean	<	10.0 Gy(RBE)	5			
**Stomach/DD:**	2.0 Gy	Max	<	20.0 Gy(RBE)	5	10		
		V20	<	15.0%	5			
**Large intestine:**	4.0 Gy	Max	<	20.0 Gy(RBE)	5	10		
		V35	<	10.0%	5			
**Skin:**	3.0 Gy	Max Isodose	<	50.0%	5	5		
**Interstitial Tissue:**	3.0 Gy							

DD = Duodenum, OAR = Organ at risk, VX = Volume being irradiated with ≥ X Gy

### Adaptions in the Treatment Planning System and Optimization

Biological plan optimization was based on specific tissue parameters that were characterized by the LEM and underlying αβ-values [13]. Overlapping structures are common in the underlying structure set–e.g. the PTV takes into account the microscopic tumor extension and setup inaccuracies which leads to an overlap with nearly located OARs (OAR intersection in Table 1). For the project of biological plan optimization, it was crucial to assign a discrete αβ-value for the algorithm which obviously represents a conflict in overlapping areas where different αβ-values were possible due to the overlapping structures.

**Fig 2:**
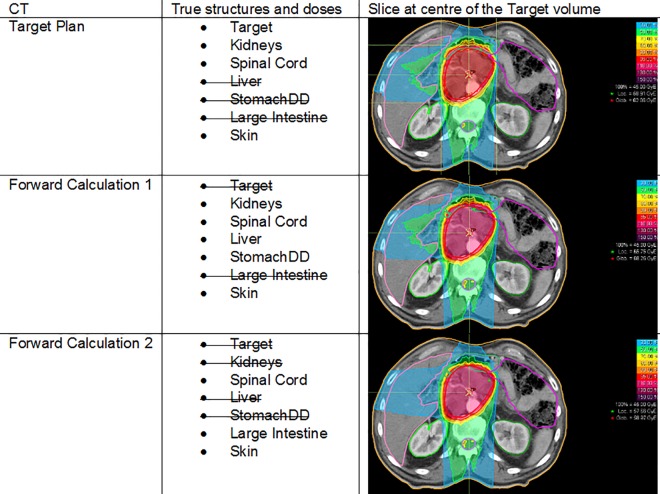
Optimization Process: Case 5, FS 5. Optimization process consisting of up to 3 steps: target plan optimization and 2 forward calculations.

With biological optimization not being possible for overlapping biological cell types forward calculations were performed to resolve this conflict: in the case of three different overlapping biological cell types, there are three identical CTs needed for treatment planning: The first CT with its structure set is adapted to the target volume, the other structures are subtracted. This leads to a target volume which is only defined by a single αβ-ratio of 5 Gy. Afterwards the plan optimization was conducted on this adapted structure set (including the target volume and the adapted OARs), and forward calculations of the ion counts on the copies allowed to extract the true doses of the unsubtracted OARs. For each plan, the true doses have to be collected from up to 3 different DVHs and pictures (Fig 2).

### Treatment Plan Evaluation

A total of 60 DVHs were evaluated–taking into account 5 FS with 25 treatment plans and 35 forward calculations. The DVH parameters are listed in S1 Table.

All plans of each case were compared with each other by the use of a customized index consisting of Target and OAR criteria (Table 2). These criteria are significant for toxicity and derived from daily clinical practice, and therefore the basic constraints of our plan optimization. On the basis of these criteria, points are allocated to the plans, thus making plan comparison possible.

The OAR criteria of the spinal cord, the kidneys and liver are equal; stomach/duodenum and large intestine 33% less; skin 66% less. So, the criteria’s value of spinal cord, kidneys and liver is increased–these OARs have more maximum points than the other OARs. This is due to the fact, that our field setups prefer beams crossing these organs—the mentioned organs are consequently exposed to higher doses and therefore of higher relevance.

With regard to each criterion, the plans of one case are compared with each other. The plans fulfilling the criterion are serialized. The plan with the best DVH parameters gets the maximum number of points (listed in Table 2). The other plans receive less points in linear intervals. If a plan does not meet the criterion, points are deducted in the same linear intervals: For example, the five plans’ point grading of the only spinal cord-criterion: 15-12-9-6-3 –minus points are distributed, starting with -3. With the kidney having three criteria and the OARs spinal cord and each kidney being of equal value, each criterion has a maximum value of 15/3 = 5. The sum of all points of all target criteria together makes the index “Target criteria” and the sum of all points of all OAR criteria together makes the index “OAR criteria”. “Target criteria” and “OAR criteria” together make the index “Cumulative Criteria”. The Index evaluation is listed in S2 Table.

## Results

### DVH Evaluation: For Case 5

As an example we describe the true DVH parameters of case 5.

For FS1 the target volume getting less than 95% of the prescribed dose (1-V42.75) is 0.1% and the volume getting 50 Gy(RBE) is 0.7%. The maximum dose (D_max_) in the spinal cord is very high with 51.5 Gy(RBE). The mean dose (D_mean_) in the right and left kidney is 3.4 Gy(RBE) and 0.2 Gy(RBE) respectively. The liver has a low D_mean_ of 1 Gy(RBE).

1-V42.75 in the target is 1.2% for FS2 and 1.9% for FS3. V50 is 0.4% for FS2 and 0.2% for FS3. The D_max_ in the spinal cord is lower than with FS1 (41 Gy(RBE) for FS2, 36 Gy(RBE) for FS3). With these field configurations travelling through the kidneys, the D_mean_ is relatively high: in the right kidney 4.5 Gy(RBE) and 4.9 Gy(RBE) for FS2 and 3, and in the left kidney 5.4 Gy(RBE) and 8.8 Gy(RBE) for FS2 and 3. The D_mean_ in the liver is 1.1 Gy(RBE) for FS2 and 5.5 Gy(RBE) for FS3.

The criterion 1-V42.75 < 5% cannot be met by FS4, but is met in FS5 with 3.6%. V50 is in both FSs on a very low level of 0.1% and 0.3%. The doses in the OARs are relatively low. The D_max_ in the spinal cord is 32.1 Gy(RBE) for FS4 and 32.5 Gy(RBE) for FS5.The D_mean_ in the kidneys is for FS4 1 Gy(RBE) in the right and 0.6 Gy(RBE) in the left one—similarly for FS5. The liver criterion is met with both FS4 and FS5.

### Index Evaluation (Fig 3)

With regard to the target criteria, the single posterior field achieves the highest scores with a mean of 23 points. But for the OAR criteria the field setups made up of 3 fields show the highest scores. FS4 achieved a mean score of 35 points. FS1 has a mean score of only 23 points, but the point dispersion is very small with 18–26 points. On top of that, for case 3 the results of FS1 are equal to those of FS4 and 5. The cumulative criteria confirm this tendency. But the high points of FS1 for the target criteria lead to an equalization of the cumulative score, especially in the cases 1,2, and 4. For the cases 3 and 5 the score shows high points with FS1.

**Fig 3:**
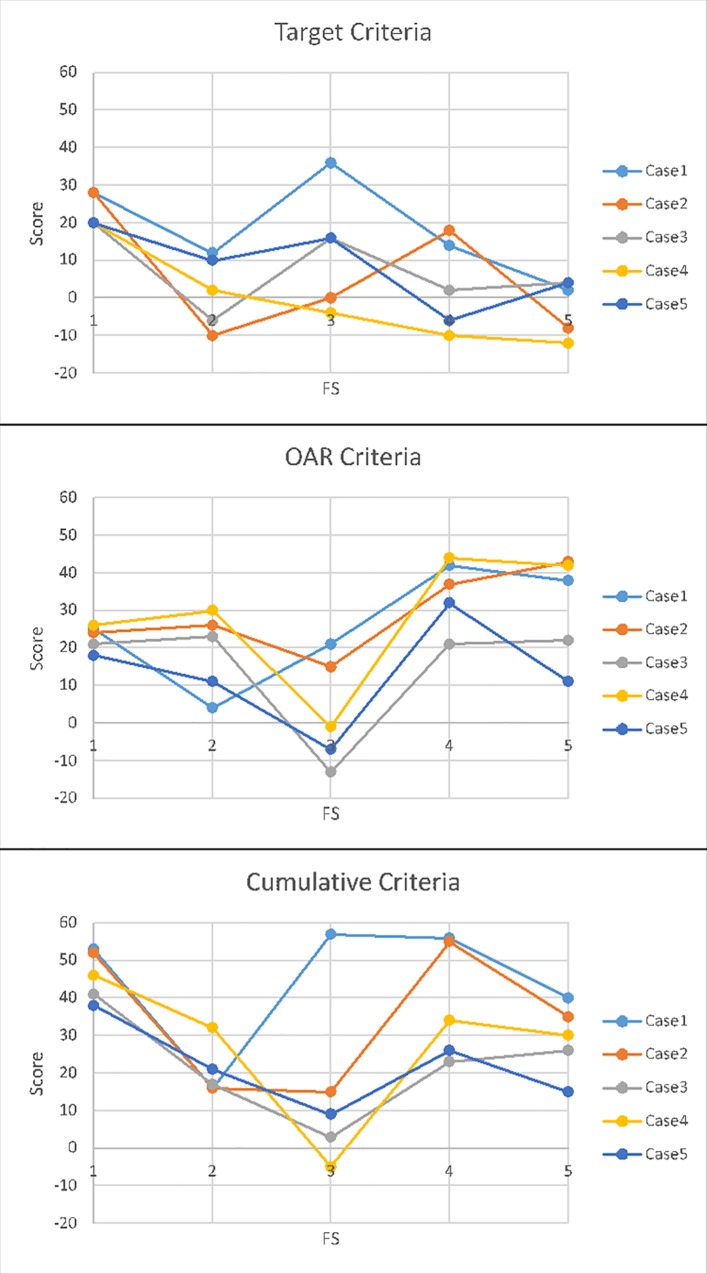
Index Evaluation. Comparison of the plans by the three indices. Target Criteria, OAR Criteria, and Cumulative Criteria.

In summary, the dose coverage in the target is weaker in cases of field setups with weighting factors, but the OAR sparing is ensured, especially with regard to the spinal cord. Nevertheless, both in the presented case 5 and in case 3 FS1 achieves good results in our index—these cases offer a topographic proximity of the target to the OARs and the OARs to each other (Table 1). Despite of these results, one has to be clear about the fact, that FS1 exceeds the permitted maximum dose in the spinal cord, with the highest dose of 51.5 Gy(RBE) in the presented case 5. On top of that, one has to take into account, that with regard to the cases 1, 2, and 4 FS4 and 5 get very good results—the OAR sparing and dose coverage in the target is even better. With regard to the isodoses reaching up to the skin, FS4&5 are the only ones to be able to meet the criteria (<50% Isodose) for all the cases except case 5.

### Evaluation of dose profiles (Table 3)

**Fig 4:**
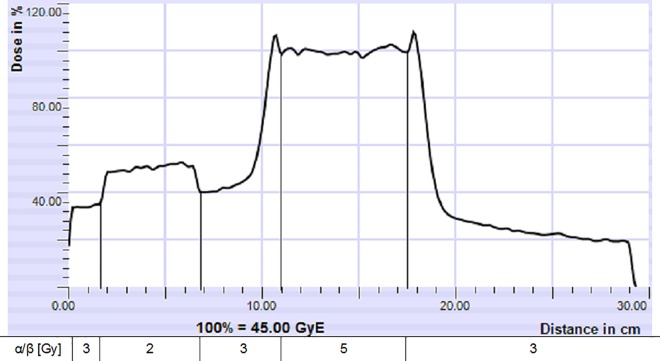
Dose profile in the beam channel: Case 4, FS 4, 270°. Demonstration of the dose profile from the proximal to the distal part of the beam channel.

As shown in Fig 4, the RBE weighted dose increases at the proximal and distal border of the target due to αβ-gradients. Evaluating these dose peaks for each FS and beam angle on height of the target’s volumetric center, FS1 shows the highest peaks in the normal tissue surrounding the tumor. On top of that, the dose peak in the distal beam channel is higher than proximal of the target.

**Table 3:** FS Evaluation: Dose profile.

Field setup	Beam angle	Mean dose peak [% of 45Gy(RBE)]	
		proximal	distal
FS1	185°	114	122
FS2	165°	114	108
	195°	113	107
FS3	155°	106	108
	245°	103	111
FS4	180°	101	104
	270°	104	109
	90°	109	106
FS5	180°	105	107
	270°	100	109
	0°	107	105

FS = Field setup

## Discussion

As hepatobiliary and pancreatic malignancies are surrounded by highly radiosensitive normal tissues, particle therapy is a promising technique with regard to radiotherapy of LAPC [19]. As pancreatic cancer and the OARs are characterized by different radiobiological sensitivities, the purpose of the study was to expand our previous study on a general field setup evaluation[10]. In our former study an αβ-ratio of 2 Gy was allocated to all tissues, representing the implemented workflow at our institution. We have now integrated the tissues’ specific αβ-ratios in our TPS, in order to deliver insight into the biological based dose exposure of normal and target tissues, and to expand the treatment planning process of carbon ion radiotherapy. In contrast to our former study, treatment planning had to be expanded by forward calculations in order to deal with overlapping structures, that is to say overlapping αβ-ratios.

To ensure a highly critical view on this setup, we integrated the αβ-ratios of tissue-specific long term sequelae by purpose. Integrating an αβ-value of 5 Gy for pancreatic cancer, a higher amount of absolute dose has to be deposited in the target (in contrast to an αβ-value of 2 Gy in our former study) [10, 14]. This results in very steep biological gradients, thus leading to high doses in the OARs. With the αβ-ratio being smaller in the surrounding tissue, the RBE dose exceeds the prescribed dose outside of the target volume. Therefore, this study has to be seen as a worst case scenario.

In summary, a three-field setup achieved good score values, but a one-field arrangement showed comparable or even better results in cases with topographic proximity of normal to target tissues. Moreover, the field setup with only one field has little score variations. But, regarding the prescribed target dose of 45 Gy(RBE) and the achieved maximum point dose to the spinal cord with values exceeding 110% in field-setup 1 (single posterior field), this field configuration violates the general QUANTEC (Quantitative Analyses of Normal Tissue Effects in the Clinic) dose constraints of 50 Gy(RBE) [20]. In a previous work of our group plan optimization was performed with a homogeneous αβ-value of 2 Gy and FS1 did not result in dose excess to the spinal cord—maximum point doses were below 45 Gy(RBE) in all scenarios [10]. Nonetheless, tolerance doses of normal tissues are extrapolated from photon-based data, which is why tolerance doses of the OARs in ion beam therapy have to be critically reevaluated.

The three fields setups are able to reduce the maximum dose in the spinal cord and other OARs despite the large αβ-gradient. But, weighting factors in the field setups with three fields result in poorer dose coverage of the target. Another advantage of the FS made up of three fields is obvious with regard to our dose profile evaluation. The dose peaks both in the proximal and distal beam channel at the αβ-border are the highest with FS1. This steep RBE-increase at the distal end of the SOBP results in unexpected high doses, for example in the stomach which is located ventrally of the pancreas. FS1 may have its advantages in case of difficult anatomical conditions, but it has to be critically evaluated.

Nevertheless, the current TPS version is based on LEM I—the dose in the proximal part of the beam channel is rather overestimated and the dose in the target itself is underestimated as recently demonstrated with an improved version of the local effect model (LEM IV) [21]. So our worst case scenario is also aggravated by the TPS itself.

With biological based treatment planning in particle radiotherapy, we get the chance to evaluate dose exposure of normal tissues with much more precision. But we have to be clear about the fact, that precision in daily clinical practice is not only ensured by precise DVHs, but by handling intra- and inter-fractional variations. Especially particle therapy of abdominal organs needs to take organ motion and bowel gas movement into account [8, 9].

According to Kumagai and colleagues, we restricted three fields setups by weighting factors for anterior-posterior and lateral beams [8]. Due to gastrointestinal gas bubbles, these beams seem to have the highest dose affections. Corresponding to Kumagai et al. and our former study, this study points to more robustness of a single posterior field compared to three-field arrangements [8, 10]. Breathing induced motion causes relevant changes in the beam path and can perhaps be met with gating, such as irradiation only during expiration–Taniguchi and colleagues showed a decreasing dose to the OARs during expiration compared to inspiration [22]. On top of that, interplay effects may be caused by interactions of beams with organ motion–unwanted normal tissue exposure can arise. But a study about ion beam irradiation of liver tumors by Richter et al. could show, that inhomogeneity by interplay effects may be reduced by fractionation [23].

So, intra- as well as interfractional changes are described but not totally understood. Especially in scanned ion beam treatment, re-planning scenarios are needed as slight changes probably lead to significant dose variations [8, 9, 22, 24]. Moreover, our references are mainly experiences from Japanese institutions in ion beam therapy of pancreatic cancer. In this setting, irradiation was performed by the scattered ion beam therapy. In our department, investigations in robustness of scanned ion beam therapy of LAPC have been made by Batista et al. Their data support our results, showing high robustness of a single posterior field [25].

Nonetheless, our study was able to expand our first study on treatment planning of particle therapy of LAPC, thus leading to treatment planning including the tissues’ specific αβ ratios. Particle therapy of pancreatic cancer is possible by the use of all 5 field setups, but distinct topographical conditions and the biologically weighted dose exposure to the normal tissues should be taken into account.

## Conclusion

Integrating specific αβ-ratios for all affected tissues is the ultimate challenge for biological-based treatment planning, especially in case of carbon ion therapy. A field setup with one single posterior field achieves good results, especially in cases of topographic proximity of organs at risk to the target volume, but leads to probably unacceptable high maximum doses to the spinal cord. So, field setups with multiple fields seem to be more adequate in case of LAPC being surrounded by highly radiosensitive OARs.

## Supporting Information

S1 Table Dataset DVH. (XLSX)

S2 Table Dataset Index. (XLSX)
